# Editorial: Sharpen the tools to decipher cell-cell communication better

**DOI:** 10.3389/fimmu.2026.2021485

**Published:** 2026-10-20

**Authors:** Qingfei Pan, Koon-Kiu Yan, Suoqin Jin

**Affiliations:** 1 Department of Computational Biology, St. Jude Children's Research Hospital, Memphis, TN, United States; 2 Department of Bone Marrow Transplantation and Cellular Therapy, St. Jude Children's Research Hospital, Memphis, TN, United States; 3 School of Mathematics and Statistics, Wuhan University, Wuhan, China

**Keywords:** cell-cell communication, ligand-receptor interactions, single-cell genomics, spatial omics, multimodal integration, functional validation, causal validation

Cell-cell communication (CCC) orchestrates virtually every aspect of tissue biology, from development and homeostasis to immune responses and disease progression. Recent advances in single-cell and spatially resolved technologies have transformed our ability to dissect these interactions at unprecedented resolution. Yet significant challenges remain. Conventional single-cell RNA sequencing (scRNA-seq) approaches often disrupt tissue architecture, computational methods may infer interactions without confirming physical proximity, and the biological and clinical relevance of CCC frequently requires orthogonal validation. Against this backdrop, the Research Topic “*Sharpen the tools to decipher cell-cell communication better*” was established to highlight methodological and biological advances that improve our capacity to identify, contextualize, and interpret cellular interactions across health and disease.

The four contributions assembled in this Research Topic illustrate the complementary directions in which the field is advancing: development of novel computational frameworks, integration of multimodal spatial technologies, rigorous validation of communication networks, and translation of these discoveries into clinically relevant biological insights.

A major limitation of current CCC inference approaches is the difficulty of capturing direct physical interactions among cells. Addressing this challenge, Hameed et al. developed ULMnet, a computational framework that exploits multiplets in scRNA-seq data to infer physical cell-cell interaction networks. Rather than treating doublets and multiplets solely as technical artifacts, the authors recognized that many originate from physically associated cells that remain attached during tissue processing. ULMnet employs univariate linear models to identify and deconvolute these multiplets, enabling reconstruction of physical interaction networks. Importantly, the approach was validated using experimentally defined cell pairs, partially dissociated tissues, and matched spatial transcriptomic datasets, demonstrating its capacity to recover biologically meaningful tissue organization and cellular neighborhoods. This work highlights an emerging shift in CCC analysis from purely ligand-receptor inference toward reconstruction of physical cellular architectures.

While methodological innovation is essential, experimental validation remains equally important. In lung adenocarcinoma, Luo et al. integrated scRNA-seq, spatial transcriptomics, and immunofluorescence analyses to characterize changes in cell-cell communication during histological progression from lepidic to solid tumor patterns. Their study revealed that immune-suppressive communication networks progressively intensify during tumor evolution. In particular, the TIGIT-NECTIN2, PVR-TIGIT, and PD-1/PD-L1 axes emerged as critical pathways associated with immune evasion, macrophage recruitment, and T-cell exhaustion. By combining computational inference with spatial validation, the authors demonstrated the co-localization of receptor-ligand pairs within tumor tissues and linked these interactions to clinical outcomes and therapeutic responsiveness. The study underscores how multimodal validation can transform communication predictions into biologically and clinically meaningful mechanisms.

A complementary example of translational application is provided by Fu et al. in clear cell renal cell carcinoma (ccRCC). Integrating scRNA-seq and spatial transcriptomics, the authors identified epithelial-cell-associated prognostic signatures and generated a robust risk stratification model based on seven genes. Beyond prognostic modeling, the study uncovered ATP1A1 as a potential mediator linking vascular biology and immune regulation. Functional validation demonstrated that ATP1A1 inhibited endothelial cell migration and angiogenesis while promoting M1 macrophage polarization. These findings illustrate how integrating communication-aware spatial analyses with functional studies can reveal biomarkers and therapeutic targets that connect cellular interactions to disease outcomes. More broadly, the work highlights how CCC research is increasingly moving from descriptive network reconstruction toward clinically actionable discoveries.

Extending the scope of the Topic beyond oncology, Sisó et al. applied a comprehensive spatial multi-omics framework to rheumatoid arthritis (RA), integrating multiple spatial transcriptomic platforms, spatial proteomics, multiplex immunohistochemistry, and computational pathology. Their study represents an important contribution to the understanding of tissue architecture in chronic inflammatory disease. Cross-platform analyses demonstrated substantial concordance in cellular composition and localization while highlighting the complementary strengths of different spatial technologies. Most notably, the authors identified a previously unrecognized laminar organization of fibroblast subtypes within the RA synovium. Through single-cell spatial analyses, they revealed distinct fibroblast populations occupying lymphoid, vascular, and stromal niches and demonstrated how these subtypes form disease-associated cellular neighborhoods. By linking spatial organization to pathway activities and tissue microenvironments, the study illustrates how advanced spatial multi-omics approaches can uncover layers of cellular communication and organization that are inaccessible to dissociation-based methods.

Taken together, these four studies illustrate several important trends shaping the future of cell-cell communication research. First, the field is increasingly moving beyond cataloging ligand-receptor interactions toward understanding cellular interactions within their spatial and physical contexts. Physical proximity, tissue architecture, and cellular neighborhoods are emerging as critical determinants of communication fidelity. Second, multimodal integration is becoming an important frontier. The combination of scRNA-seq, spatial transcriptomics, proteomics, imaging technologies, and computational modeling provides a more comprehensive and reliable view of cellular interactions than any single modality alone. Third, validation is receiving greater emphasis. Studies in this Topic consistently demonstrate the value of combining computational predictions with spatial, histological, or functional evidence. Finally, CCC research is becoming increasingly translational, with discoveries being linked to prognosis, therapeutic response, immune regulation, and disease mechanisms.

Despite remarkable progress, significant challenges remain. First, current spatial transcriptomics technologies vary substantially in spatial resolution, gene coverage, and measurement sensitivity, creating challenges for cross-platform analyses and method generalization. This field would benefit from unified, robust, and scalable frameworks capable of systematically inferring spatially resolved CCC at single-cell resolution across diverse platforms. Second, comparative and longitudinal analyses of CCC across biological states, developmental stages, disease progression, and treatment responses remain underexplored. Such analyses can reveal how communication networks are dynamically rewired over time and across contexts. Furthermore, translating inferred interactions into functional and causal mechanisms remains an important frontier. Emerging technologies such as spatially-resolved CRISPR screens may enable direct testing of the causal effects of specific L-R interactions.

The studies collected in this Research Topic exemplify the maturation of the CCC field from computational inference toward mechanistic understanding. By sharpening both computational and experimental tools, CCC research will move beyond descriptive analysis toward predictive and intervention-oriented modeling, enabling the prediction of how intercellular communication responds to genetic, pharmacological, or environmental perturbations in cancer, autoimmune disease, and beyond. As technologies continue to evolve, such integrative approaches will be instrumental in translating cellular interaction maps into biological understanding and ultimately into improved diagnostic and therapeutic strategies.

